# Adherence to Cancer Screening Guidelines and Predictors of Improvement Among Participants in the Kansas State Employee Wellness Program

**DOI:** 10.5888/pcd10.120212

**Published:** 2013-07-11

**Authors:** Siu-kuen Azor Hui, Kimberly K. Engelman, Theresa I. Shireman, Edward F. Ellerbeck

**Affiliations:** Author Affiliations: Kimberly K. Engelman, Theresa I. Shireman, Edward F. Ellerbeck, Department of Preventive Medicine and Public Health, University of Kansas Medical Center, Kansas City, Kansas.

## Abstract

**Introduction:**

Employee wellness programs (EWPs) have been used to implement worksite-based cancer prevention and control interventions. However, little is known about whether these programs result in improved adherence to cancer screening guidelines or how participants’ characteristics affect subsequent screening. This study was conducted to describe cancer screening behaviors among participants in a state EWP and identify factors associated with screening adherence among those who were initially nonadherent.

**Methods:**

We identified employees and their dependents who completed health risk assessments (HRAs) as part of the Kansas state EWP in both 2008 and 2009. We examined baseline rates of adherence to cancer screening guidelines in 2008 and factors associated with adherence in 2009 among participants who were initially nonadherent.

**Results:**

Of 53,095 eligible participants, 13,222 (25%) participated in the EWP in 2008 and 6,205 (12%) participated in both years. Among the multiyear participants, adherence was high at baseline to screening for breast (92.5%), cervical (91.8%), and colorectal cancer (72.7%). Of participants who were initially nonadherent in 2008, 52.4%, 41.3%, and 33.5%, respectively, became adherent in the following year to breast, cervical, and colorectal cancer screening. Suburban/urban residence and more frequent doctor visits predicted adherence to breast and colorectal cancer screening guidelines.

**Conclusion:**

The effectiveness of EWPs for increasing cancer screening is limited by low HRA participation rates, high rates of adherence to screening at baseline, and failure of nonadherent participants to get screening. Improving overall adherence to cancer screening guidelines among employees will require efforts to increase HRA participation, stronger interventions for nonadherent participants, and better access to screening for rural employees.

## Introduction

Employee wellness programs (EWPs) are organized, employer-sponsored health promotion and chronic disease prevention programs that offer annual free health risk assessments (HRAs) to employees and their dependents who are enrolled in employer-sponsored health plans. Based on the HRA results, personalized risk feedback and preventive care recommendations are provided to voluntary participants. During the past 40 years, EWPs have proliferated as employers try to recoup greater value from their health-related expenditures. Their goals with EWPs are to 1) improve employee health, productivity, and job satisfaction and 2) reduce health care costs associated with chronic diseases (1). EWPs are offered to employees and their dependents who purchase their health insurance plans through their employers. Several studies have shown EWPs to be effective in preventing cardiovascular diseases (CVD) among employees (2–5), but their value in cancer prevention and control is less well established.

Screening for breast, cervical, and colorectal cancer can reduce illness and death from these diseases, but adherence to cancer screening guidelines remains suboptimal (6,7). Because a large proportion of adults in the United States are employed, worksites provide an opportunity to implement interventions to improve cancer screening among employees (8). Promotion of cancer screening among employees can be highly cost-effective from the employer’s perspective because of medical and disability savings from early detection of cancer (9). Given the potential of EWP-based interventions to increase cancer screening (8), the CEO Roundtable on Cancer established standards (10) for the early detection of cancer through EWPs. These standards describe health plan benefits, education programs, and various cancer screening worksite interventions that can improve adherence to cancer screening recommendations among EWP participants. However, as of January 14, 2013, only 137 organizations were accredited with the CEO Cancer Gold Standard; most organizations provide markedly fewer cancer screening interventions for EWP participants.

EWPs that incorporate cancer screening typically assess cancer screening behavior on an annual HRA questionnaire and provide full cost coverage of relevant breast, cervical, and colorectal cancer screening tests for employees. The annual HRA is commonly administered via the Internet, and it collects self-reported health-risk data by asking questions about risk factors for common health problems, including cancer. After completing the HRA, participants receive a personalized feedback report on their health-risk profile and preventive care recommendations directed toward specific identified health risks. Sometimes, participants are given referrals to programs or providers that can address their specific risks. The HRA is the critical gateway to a broader EWP, and a recent systematic review by the Task Force on Community Preventive Services found strong evidence that use of HRAs with personalized feedback combined with additional worksite interventions can improve employees’ health outcomes (4,11).

However, little is known about what happens to HRA participants who are initially nonadherent to cancer screening guidelines after they have received their personalized risk feedback report. Although previous research found that adherence to cancer screening guidelines is predictable on the basis of sociodemographic variables (eg, age, education, income) (12–14), health behavior model constructs (eg, knowledge, perceived risk, perceived barriers) (12,15,16), and engaging in other health behaviors (eg, not smoking, not being overweight) (17,18), there has been little study of predictors of adherence within the context of EWPs. This study fills in the knowledge gap of cancer screening adherence among EWP participants by examining overall participation in the EWP, changes in screening status, and factors associated with participants becoming adherent to cancer screening guidelines.

## Methods

This study used 2008 and 2009 HRA data from the Kansas EWP. The data were obtained through a data use agreement between the University of Kansas Medical Center and the Kansas Health Policy Authority. Data included the basic personnel data of all eligible participants and complete responses of all HRA participants. Each person represented in these files had a unique numerical identifier. Because the coding of the numerical identifier was unknown to the authors, these data were not considered as personally identifiable, and the Human Subjects Committee at the University of Kansas Medical Center approved the study.

### Participants

The participants of this study were Kansas state employees and their dependents who enrolled in the Kansas state employee health plan and completed a standard HRA in 2008 and 2009. Among the 6,205 employees and their dependents who participated in the HRA in both years, only those who were eligible for the recommended cancer screening but who were nonadherent in 2008 (N = 1,225) were included in this study (Table 1). The eligible, nonadherent populations were defined according to the clinical preventive services guide issued by the US Preventive Services Task Force (USPSTF) in 2009 (19). 

**Table 1:** Demographic Characteristics of Participants (N = 1,225) of an Employee Worksite Wellness Program Who Were Nonadherent to Cancer Screening Guidelines in 2008, Kansas, 2009

Demographic Variable	Breast Cancer Screening	Cervical Cancer Screening	Colorectal Cancer Screening

	(n = 210)	(n = 320)	(n = 695)

	No. (%) Who Were Nonadherent in 2008		
**Age, y**			
20–29	—	21 (6.6)	—
30–39	—	37 (11.6)	—
40–49	137 (65.2)	102 (31.9)	—
50–59	64 (30.5)	143 (44.7)	588 (84.6)
≥60	9 (4.3)	17 (5.3)	107 (15.4)
**Race/ethnicity^a^**			
White	188 (89.5)	288 (90.0)	651 (93.7)
African American	8 (3.8)	11 (3.4)	19 (2.7)
American Indian/Native American	11 (5.2)	11 (3.4)	14 (2.0)
Asian/Pacific Islander	8 (3.8)	13 (4.1)	18 (2.5)
Hispanic	5 (2.4)	7 (2.2)	9 (1.3)
**Education**			
Some high school or less	0	1 (0.3)	7 (1.0)
High school graduate	31 (14.8)	52 (16.3)	104 (15.0)
Some college	66 (31.4)	101 (31.6)	245 (35.3)
College graduate	80 (38.1)	107 (33.4)	202 (29.1)
Postgrad/Professional degree	33 (15.7)	59 (18.4)	137 (19.7)
**Income, $**			
0-20,000	9 (4.3)	20 (6.3)	40 (5.8)
20,001–35,000	85 (40.5)	134 (41.9)	214 (30.8)
35,001–55,000	78 (37.1)	105 (32.8)	256 (36.8)
55,001–85,000	12 (5.7)	25 (7.8)	94 (13.5)
85,001–100,000	1 (0.5)	3 (0.9)	12 (1.7)
≥100,001	2 (1.0)	4 (1.3)	7 (1.0)
Do not wish to share income/missing	23 (10.9)	29 (9.0)	72 (10.4)
**Work type**			
Operator/assembly/labor/construction	2 (1.0)	3 (0.9)	33 (4.7)
Production/craftsman/machinist/carpenter	0	0	17 (2.4)
Service occupation/janitorial	10 (4.8)	12 (3.8)	40 (5.8)
Clerical and administrative support	76 (36.2)	121 (37.8)	189 (27.2)
Sales	0	0	7 (1.0)
Technical support	9 (4.3)	13 (4.1)	49 (7.1)
Professional	81 (38.6)	123 (38.4)	221 (31.8)
Executive or manager	25 (11.9)	37 (11.6)	110 (15.8)
Missing	7 (3.3)	11 (3.4)	29 (4.2)

Abbreviation: — , not applicable.

a Race/ethnicity categories are not mutually exclusive.

### Measures

The screening adherence measures used were the same on the 2008 and 2009 HRA questionnaires. Initial screening nonadherence status was derived from the 2008 HRA responses, and screening adherence status was derived from the 2009 HRA responses. Breast cancer screening nonadherence was defined as women aged 40 years or older who reported having their last mammography “more than 2 years ago” or “never”; adherence was defined as the eligible women having their last mammography “less than 1 year ago” or “1 to 2 years ago.” Cervical cancer screening nonadherence was defined as women aged 21 to 65 years who reported having their last Papanicolaou (Pap) test “more than 2 years ago” or “never”; adherence was defined as the eligible women having the last Pap test “less than 1 year ago” or “1 to 2 years ago.” Although USPSTF recommends cervical cancer screening only every 3 years for women in this age group, there was no response choice of “more than 3 years ago” on the 2008 HRA, so we could not determine the number of women who were nonadherent. Colorectal cancer screening nonadherence was defined as men or women aged 50 years or older who responded no to having had a fecal occult blood test (FOBT) in the past 12 months, sigmoidoscopy in the past 5 years, and colonoscopy in the past 10 years; screening adherence was defined as responding yes to 1 of the 3 questions.

The potential predictors of these outcomes were derived from the 2008 HRA answers. Education was dichotomized into “some college or lower” or “college graduate or higher,” income was dichotomized into $0 to $35,000 or over $35,000), living setting was dichotomized into “rural” or “suburban or urban,” and job ranking was dichotomized into “senior” (ie, professionals and executive or managers) and “junior” (all others). Perceived health risk was measured on a 5-point Likert scale by the question, “Compared to others like me, my overall risk of getting an illness or disease is . . . much higher, higher, about the same, lower, or much lower.” This variable also was dichotomized. Participants who answered “about the same, lower, or much lower” were regarded as perceiving their health to be “about the same or healthier” than that of their peers, and those who answered “higher” or “much higher” were regarded as perceiving their health to be “less healthy” than that of their peers. Number of visits to a doctor’s office or clinic in the preceding year was dichotomized into “0 or 1” and “2 or more.”

A healthy lifestyle index was created to categorize participants into 2 groups in terms of health behaviors. The “healthy” group was coded as participants who reported not smoking, not overusing alcohol, having a body mass index below 25 kg/m^2^, engaging in physical activities of moderate to vigorous intensity for at least 30 minutes on 3 days per week, and eating at least 2 cups of fruit and 2.5 cups of vegetables each day. Alcohol overuse was defined as having an alcoholic drink 6 to 7 days per week and having 2 or more drinks on each day for men or 1 or more drink on each day for women (20). The definitions of health behaviors related to physical activity and fruit and vegetable intake were based on the US Department of Health and Human Services guidelines (21,22). 

### Procedure

An Internet-based portal for the Kansas State EWP was open during March to September in 2008 and 2009 for eligible participants to log on and complete their HRA. Because some questions on the HRA asked for clinical data, eligible participants were recommended to attend their worksite biometric screening first and then enter the data in HRA questionnaire. However, participants also could enter their clinical data obtained from their most recent doctor’s visit. As an incentive, participants received a $50 gift card if they completed both the HRA and the worksite screening.

Immediately after completing the online HRA, participants received their personalized disease risk report and preventive care recommendations electronically. Although questions were asked about participants’ last time of relevant cancer screening tests, the HRA personalized disease risk feedback report in 2008 did not include the participant’s cancer screening status and recommendations. All state employee health plans in 2008 and 2009 provided full coverage of the cost of all eligible cancer screening tests.

### Statistical analyses

Chi-square tests were used to examine bivariate relationships between each cancer screening adherence outcome and each baseline predictor. Odds ratios (ORs) were calculated to identify significant associations between the hypothesized predictors and cancer screening adherence. Significant bivariate predictors were analyzed by using multivariate logistic regression to investigate whether they independently predicted the outcome.

## Results

In our sample, HRA participation rates were 22% in 2008 and 15% in 2009. Among the 53,095 total health plan enrollees in 2008, 13,222 (25%) were HRA participants in 2008 and 6,205 (12%) of them also participated in 2009. Among these participants, most were adherent in their cancer screenings in 2008 (Figure). In our sample, 92.5% of eligible women were adherent to breast cancer screening, 91.8% of eligible women were adherent to cervical cancer screening, and 72.7% of eligible participants were adherent to colorectal cancer screening in 2008. Among the 210 women who were nonadherent to breast cancer screening in 2008, 110 (52.4%) became adherent in 2009. Among 320 women who were nonadherent to cervical cancer screening in 2008, 132 (41.3%) became adherent in 2009. Among 695 men and women who were nonadherent to colorectal cancer screening in 2008, 233 (33.5%) became adherent in 2009.

**Figure:**
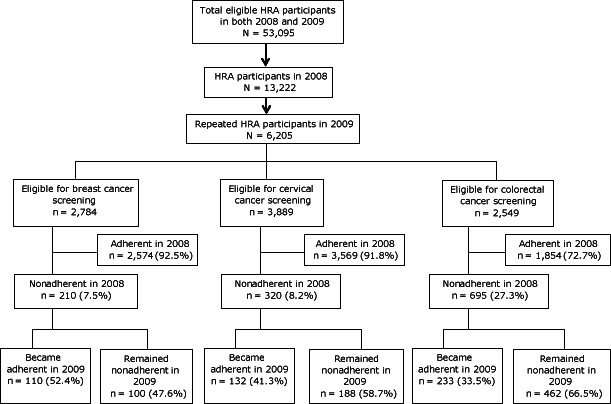
Flow chart depicting inclusion process of participants who completed health risk assessments (HRAs) and their cancer screening adherence behaviors, Kansas, 2008 and 2009

Among women who were initially nonadherent to breast cancer screening, those who lived in suburban or urban areas were more likely to become adherent the following year than those who lived in rural areas (OR = 1.99, *P* = .03) (Table 2). Among men and women who were initially nonadherent to colorectal cancer screening, significant bivariate predictors of improvement were suburban/urban residence (OR = 1.57, *P* = .01) and 2 or more doctor’s visits during the previous year (OR = 1.43, *P* = .04). Further multivariate logistic regression modeling showed that suburban/urban residence remained as a significant independent predictor of the improvement in colorectal cancer screening (OR = 1.54, *P* < .05), while number of doctor’s visits became nonsignificant (OR = 1.39, *P* = .056) as an independent predictor. No significant predictors were found for improvement in cervical cancer screening adherence.

**Table 2:** Factors Associated With Adhering to Cancer Screening Guidelines in 2009 Among Eligible Individuals Who Were Nonadherent in 2008, Employee Worksite Wellness Program, Kansas

Baseline Predictors in 2008	Breast Cancer Screening Adherent		Cervical Cancer Screening Adherent		Colorectal Cancer Screening Adherent	
	N (%)	OR (95% CI)	N (%)	OR (95% CI)	N (%)	OR (95% CI)
**Sex**						
Male	—	—	—	—	237 (32.9)	1 [Reference]
Female	—	—	—	—	458 (33.8)	1.04 (0.75–1.46)
**Education**						
Some college or lower	97 (52.6)	1 [Reference]	154 (40.9)	1 [Reference]	356 (30.3)	1 [Reference]
College graduate or higher	113 (52.2)	0.99 (0.57–1.70)	166 (41.6)	1.03 (0.66–1.60)	339 (36.9)	1.34 (0.98–1.84)
**Income, $**						
0–35,000	94 (54.3)	1 [Reference]	154 (44.8)	1 [Reference]	254 (32.3)	1 [Reference]
≥35,001	93 (55.9)	1.07 (0.60–1.90)	137 (40.1)	0.83 (0.52–1.32)	369 (35.0)	1.13 (0.80–1.58)
**Living setting**						
Rural	63 (39.7)	1 [Reference]	214 (41.6)	1 [Reference]	212 (26.9)	1 [Reference]
Suburban or urban	143 (56.6)	1.99 (1.09–3.63)^a^	100 (40.0)	1.07 (0.66–1.73)	481 (36.6)	1.57 (1.10–2.24)^b^
**Job ranking**						
Junior	97 (55.7)	1 [Reference]	149 (40.9)	1 [Reference]	335 (33.7)	1 [Reference]
Senior	106 (50.9)	0.83 (0.48–1.44)	160 (41.3)	1.01 (0.64–1.59)	331 (33.2)	0.98 (0.71–1.35)
**Perceived health risk**						
Less healthy	68 (50.0)	1 [Reference]	87 (41.4)	1 [Reference]	290 (37.6)	1 [Reference]
About the same or healthier	118 (50.0)	1.00 (0.55–1.82)	200 (40.5)	0.96 (0.58–1.61)	318 (30.5)	0.73 (0.52–1.02)
**Healthy lifestyle**						
No	180 (51.7)	1 [Reference]	274 (42.7)	1 [Reference]	596 (32.9)	1 [Reference]
Yes	30 (56.7)	1.22 (0.56–2.67)	46 (32.6)	0.65 (0.34–1.26)	99 (37.4)	1.22 (0.78–1.89)
**No. of doctor’s visits in the previous year**						
0–1	91 (49.5)	1 [Reference]	114 (41.2)	1 [Reference]	239 (28.5)	1 [Reference]
≥2	119 (54.6)	1.23 (0.71–2.13)	206 (41.3)	1.00 (0.63–1.59)	456 (36.2)	1.43 (1.02–2.00)^c^

Abbreviation: — , not applicable.

a *P* = .03.

b *P* = .01.

c *P* = .04.

## Discussion

Results of this study showed that most multiple-year health risk assessment (HRA) participants were adherent to cancer screening recommendations at baseline. This high adherence rate may be due to healthier HRA participants self-selecting to participate in HRAs more often than nonparticipants, particularly when HRA participation rates are low (23–27), which is a common phenomenon in many employee wellness programs (EWPs) (28). Further self-selection bias may have occurred among these multiple-year HRA participants. Our observed cancer screening adherence rates at baseline among these multiple-year participants were slightly higher than what we observed among the single-year participants (29). The breast cancer screening adherence rate also was substantially higher than that of the general public (92.5% vs 76%), as measured by the Centers for Disease Control and Prevention’s Behavioral Risk Factor Surveillance System (BRFSS) 2008 survey (6). Other cancer screening adherence measures used by the BRFSS survey were different and, therefore, cannot be compared directly. 

Our study demonstrates multiple levels in which an EWP may fail in its efforts to enhance cancer screening adherence. First, we noted low participation rates overall. Second, multiyear HRA participants appear to be a self-selected group of healthier workers that are less likely to need help in getting cancer screening (29). Third, among multiyear HRA participants, approximately one-half to two-thirds of those initially nonadherent to cancer screening guidelines remained nonadherent the following year. This finding is disappointing considering that these were self-selected, apparently more health-conscious employees. It is reasonable to assume that non-HRA participants would have lower overall screening rates and would be less likely to increase screening from year to year.

Of the multiyear participants who were nonadherent at baseline, our results demonstrate that rural-dwelling employees and those with less frequent visits to a doctor’s office were less likely to get screened after participating in the HRA. Access to cancer screening services and physician’s recommendation are strong predictors of adherence to cancer screening (12,16,17), and our findings likely point to these mechanisms of actions. Living in suburban/urban areas makes access to screening facilities more convenient, and more frequent encounters with doctors increases the chance of receiving reminders. This finding is consistent with previous research that rural-residing individuals experience more barriers to health care access and are less likely to receive cancer screening (30–33). This finding suggests that employees who reside in rural areas may benefit the most from worksite interventions to enhance cancer screening services but that the EWP may need to address specific barriers to accessing a provider that can provide these preventive services.

Although systematic strategies to use HRAs and EWPs to enhance cancer screening have been recommended (8), these strategies may not be implemented at most worksites or not implemented effectively. For example, one of the USPSTF recommendations for maximally effective worksite health interventions is to provide cancer screening behavior feedback, recommendations, or referrals for screening resources (4,11); however, participants in this study did not receive these interventions. Although completing the HRA itself may make particularly health-conscious employees aware of their nonadherence and serve as a cue to action to initiate screening, the HRA participants did not benefit from more direct interventions to improve their cancer screening adherence. Also, it is unlikely that smaller employers in rural communities would be able to provide permanent worksite health promotion programs. Mobile solutions or partnerships with existing rural health care providers may be required.

The strengths of this study include the use of a large state employee sample and having the complete individual-level HRA response data for 2 consecutive years. This is the first study to examine predictors of improvement in adherence to cancer screening guidelines in a population of EWP participants. One limitation of this study is the lack of a non-HRA comparison group. Other limitations were the self-report nature of the HRA responses and some HRA questions that did not match standard guidelines. Furthermore, the findings from this study cannot be generalized to all employees or EWPs.

In conclusion, EWPs offer unique opportunities to improve adherence to cancer screening guidelines among employees and their dependents. However, to maximize their public health effects, EWPs need higher HRA participation rates to reduce the healthy participants effect and improved interventions to promote cancer screening among those who have not previously had screening, including cancer screening adherence status feedback, recommendations, and screening resource information. Interventions that help employees access screening services may be particularly important for rural employees. This study demonstrates the importance and utility of tracking data in EWPs to identify opportunities for improvement in cancer screening, and it suggests the need for additional worksite-based interventions to remove barriers to cancer screening for employees living in rural areas.
